# Impact of Dietary Aromatic Amino Acids on Osteoclastic Activity

**DOI:** 10.1007/s00223-014-9878-z

**Published:** 2014-07-08

**Authors:** Mona El Refaey, Qing Zhong, Ke-Hong Ding, Xing-ming Shi, Jianrui Xu, Wendy B. Bollag, William D. Hill, Norman Chutkan, Richard Robbins, Hugh Nadeau, Maribeth Johnson, Mark W. Hamrick, Carlos M. Isales

**Affiliations:** 1 Institute for Regenerative and Reparative Medicine, Medical College of Georgia, Georgia Regents University, Augusta, GA 30912 USA; 2 Department of Orthopaedic Surgery, Medical College of Georgia, Georgia Regents University, Augusta, GA 30912 USA; 3 Departments of Medicine, Medical College of Georgia, Georgia Regents University, Augusta, GA 30912 USA; 4 Departments of Cellular Biology and Anatomy, Medical College of Georgia, Georgia Regents University, Augusta, GA 30912 USA; 5 Departments of Pathology, Medical College of Georgia, Georgia Regents University, Augusta, GA 30912 USA; 6 Departments of Physiology, Medical College of Georgia, Georgia Regents University, Augusta, GA 30912 USA; 7 Departments of Biostatistics, Medical College of Georgia, Georgia Regents University, Augusta, GA 30912 USA; 8 Charlie Norwood VA Medical Center, Augusta, GA 30912 USA

**Keywords:** Osteoclast, Amino acids, Cathepsin K, Carbonic anhydrase II, Calcitonin receptor

## Abstract

We had shown that aromatic amino acid (phenylalanine, tyrosine, and tryptophan) supplementation prevented bone loss in an aging C57BL/6 mice model. In vivo results from the markers of bone breakdown suggested an inhibition of osteoclastic activity or differentiation. To assess osteoclastic differentiation, we examined the effects of aromatic amino acids on early /structural markers as vitronectin receptor, calcitonin receptor, and carbonic anhydrase II as well as, late/functional differentiation markers; cathepsin K and matrix metalloproteinase 9 (MMP-9). Our data demonstrate that the aromatic amino acids down-regulated early and late osteoclastic differentiation markers as measured by real time PCR. Our data also suggest a link between the vitronectin receptor and the secreted cathepsin K that both showed consistent effects to the aromatic amino acid treatment. However, the non-attachment related proteins, calcitonin receptor, and carbonic anhydrase II, demonstrated less consistent effects in response to treatment. Our data are consistent with aromatic amino acids down-regulating osteoclastic differentiation by suppressing remodeling gene expression thus contributing initially to the net increase in bone mass seen in vivo.

## Introduction

Amino acids have been shown to exert direct effects on several tissues including the “amino acid-sensors” in pancreatic islets, pituitary, parathyroid gland, and liver and help regulate nutrient disposition [1]. In these tissues, amino acids have been shown to bind the extracellular calcium receptor (CaSR). The CaSR was first reported by House et al. [2] to be present in both human and mouse bone marrow cells. Bone breakdown is determined by osteoclastic activity. However, the direct effect of amino acids on osteoclasts is not known. In order to evaluate the effects of amino acids on osteoclastic development, we focused on expression of several common osteoclastic genes expressed at various levels during differentiation.

The vitronectin receptor is essential in bone remodeling as it binds to extracellular matrix proteins like osteopontin at the tri-peptide arginine-glycine-aspartic acid (RGD) recognition site and it is crucial for osteoclast polarization into clear zones and ruffled borders; two characteristic features of osteoclasts [3]. The calcitonin receptor is expressed on osteoclasts but not osteoblasts and a specific marker for osteoclast differentiation [4]. The calcitonin receptor (CTR) is a G protein-coupled receptor that is expressed at high levels by osteoclast, renal, and neural cells. It binds with highest affinity to calcitonin, which is a 32 amino acid peptide secreted by C-cells of the thyroid gland in response to elevated serum calcium levels. The main recognized action of calcitonin is to inhibit bone resorption [5].

Carbonic anhydrase II is an early marker of osteoclast differentiation and is important in bone resorption as it facilitates proton production and thus the acidic environment of the resorption lacunae. Previous work on carbonic anhydrase showed that mutation of the carbonic anhydrase II gene results in inhibition of bone resorption and osteopetrosis [6]. Studies in rat bone marrow cultures using acetazolamide, a specific carbonic anhydrase inhibitor, demonstrated that carbonic anhydrase II is crucial in proton generation in mature osteoclasts by showing a decrease in the 1,25 (OH)2D3-induced formation of multinucleated tartrate-resistant acid phosphatase (TRAP)-positive cells, in a dose-dependent manner [7].

Matrix metalloproteinase 9 (MMP-9) is also a crucial marker in bone remodeling as a recent study identified that high levels of MMP-9 have been detected in osteoclasts, MMP-9 is considered unique because of its the strong abundance and selectivity of its expression in osteoclastic cells [8, 9]. A previous study showed that MMP-9 knock-out mice displayed a bone-developmental defect, suggesting an important role for this metalloproteinase in bone turnover and remodeling [10].

Cathepsin K is considered the major cysteine protease expressed in osteoclasts [11] and has a critical role in osteoclastic bone resorption. Cathepsin K can degrade telopeptide and triple helical regions of type I collagen [12] as well as osteonectin [13]. Cathepsin K knockout mice develop osteopetrosis as a result of a deficiency in matrix degradation and decreased bone resorption [14].

We focused on aromatic amino acids (phenylalanine, tyrosine, and tryptophan) in this study as previous lab findings showed that they increased cellular activity in bone [15]. We were interested in the effect of these aromatic amino acids on osteoclastic activity through evaluation of their effects on a number of breakdown genes as vitronectin receptor, calcitonin receptor, carbonic anhydrase II, MMP-9, and cathepsin K.

## Materials and Methods

### Generation of Osteoclasts from Bone Marrow Macrophages

Male C57BL/6 mice were purchased from Harlan Laboratories (Indianapolis, In, USA). Hematopoietic stem cells were isolated from 3-month-old male C57BL/6 mice to generate macrophages and induce osteoclasts at the Georgia Regents University Stem Cell Core Facility. In brief, six mice were euthanized by CO_2_ overdose followed by thoracotomy. Whole bone marrow aspirates were flushed from femora and tibiae, both ends of the bone were cut off and long bones were flushed to flush out all bone marrow from a protocol modified from Tropel et al. [16]. Bone marrow aspirates were centrifuged at 1300 rpm for 5 min at room temperature and then cells were expanded in Alpha Modification of Eagle’s Medium (α-MEM) (cat#10-022) (α-MEM; Cellgro, Mediatech, Manassas, VA, USA) supplemented with 10 % heat-inactivated fetal bovine serum (cat#S11150) (FBS; Atlanta Biologicals, Lawrenceville, GA, USA) and 1 % penicillin-streptomycin (cat#SV30010) (Hyclone Laboratories, Inc.) for 24 h. A plastic pipette was used to collect the non-adherent cells; spun at 1300 rpm for 5 min and cells were expanded in α-MEM and macrophage-colony stimulating factor (M-CSF) (cat#315-02) (M-CSF, Peprotech Inc.) in a concentration of 50 ηg/ml for 2 days to induce macrophages. After 2 days, bone marrow macrophages were attached to the bottom of the plate; cells were collected and spun at 1300 rpm for 8 min at room temperature. 660,000 cells were seeded in each well of the six-well plate with M-CSF (cat#315-02) (M-CSF, Peprotech Inc.) (20 ng/ml) and Rank-Ligand (cat#315-11) (RANKL, Peprotech Inc.) (100 ng/ml). Different aromatic amino acid combinations were used to treat the cells during osteoclastic differentiation. After 6 days, osteoclasts were checked and confirmed with TRAP staining kit (Acid Phosphatase, Leukocyte (TRAP Kit; cat # 387, Sigma-Aldrich Co.)

### RNA Extraction and Quantitative PCR

Total cellular RNA were isolated from cells with and without aromatic amino acid treatment using TRIZOL reagent (cat#15596018) (Invitrogen) as previously described [17]. Equal amounts of total RNA (2 μg) were reverse transcribed using SuperScript III First-Strand Synthesis System (cat#18080-051) (Invitrogen) with Oligo(dT)20 as primer and 10 mM dNTP mix in a 20-μl reaction volume for 10 min at 25 °C followed by 50 min at 50 °C. The specificity was confirmed by electrophoresis of PCR products. The DNA (1 μl) was used as template for real-time RT-PCR analysis using SYBR Green Master Mix (Applied Biosystems) and a Chromo-4 real-time RT-PCR instrument (MJ Research) as previously described [17]. PCR reactions were performed in triplicate, and the levels of mRNA expression were calculated by the ΔΔCt method using 18S as an internal control (18–19). Primers for early/structural osteoclast differentiation were: vitronectin receptor, calcitonin receptor, and carbonic anhydrase II and for late/functional osteoclastic differentiation were: cathepsin K and MMP-9. Sequences for different primers for early and late differentiation markers are listed in Table 1.

**Table 1:** Primers used for real time PCR: list of primers of internal control, early and late osteoclast markers and their sequences

Primer	Gene name	Accession number	Sequence (5′→3′)	Size (bp)
Vitronectin	Vitronectin (Vtn),	NM_011707	Fwd: TGCAGCGTTCGCCCTTCCTG Rev: CCTCCTGGCTGGGTTGCTGC	110
Cathepsin K	Cathepsin K(Ctsk)	NM_007802	Fwd: CGTGCAGCAGAACGGAGGCA Rev: TAGCTGCCTTTGCCGTGGCG	95
Calcitonin receptor	Calcitonin receptor (Calcr),	NM_007588	Fwd: ACATGATCCAGTTCACCAGGCAGA Rev: AGGTTCTTGGTGACCTCCCAACTT	107
MMP9	Matrix metallo-proteinase 9 (MMP9)	NM_013599	Fwd: TGAACAAGGTGGACCATGAGGTGA Rev: TAGAGACTTGCACTGCACGGTTGA	121
Carbonic anhydrase II	Carbonic anhydrase II	M81022	Fwd: ACCACTCCGCCTCTGCTGGA Rev: ACGCCAGTTGTCCACCATCGC	144
18S	18S ribosomal RNA (Rn18 s)	NR_003278	Fwd: AGTGCGGGTCATAAGCTTGC Rev: GGGCCTCACTAAACCATCCA	134

#### In Vitro Resorption Assay

Bone marrow macrophages were cultured in 16 well BD BioCoat Osteologic slides [18–28] (BD Bioscences, San Jose, CA, USA) at a cell density of 100,000 cells/well in-MEM medium with 10 % FBS (Life Technologies, Grand Island, NY, USA), 20 ng/ml M-CSF and 100 ng/ml of RANK-L and the medium changed every 3 days. Indicated amino acids were added during the last 3 days of incubation. For each of the amino acids indicated the concentration used to stimulate the osteoclasts was double what was present in the basal-MEM medium. Baseline AA concentrations: (1) Phenylalanine-0.194 mM; (2) Tryptophan-0.049 mM; (3) Tyrosine-0.231 mM; (4) Leucine-0.397 mM; (5) Isoleucine-0.4 mM. Osteoclast identity was confirmed by TRAP staining. Resorption pits were visualized by Von Kossa staining. Pit area was estimated using NIH Image J.

### Statistical Analysis

Experiments were performed at least in triplicate from at least three independent experiments for cathepsin K, carbonic anhydrase II, vitronectin receptor, calcitonin receptor, and MMP9 expression. For real-time PCR data, the fold changes relative to control were computed using the comparative C_T_ method (ΔΔC_T_ Method) within experiment [29]. Data are expressed as the geometric mean fold change relative to control and geometric SEM. Since aromatic amino acid supplementation was hypothesized to reduce expression in these experiments, lower tail one-sample t-tests using a lognormal distribution were performed. Statistical significance was determined at alpha = 0.05 and trends were assessed between 0.05 and 0.10. No multiple testing adjustments were made [30]. Data were analyzed using SAS© 9.3 (SAS Institute, Inc., Cary, NC, USA).

## Results

### Effects of Aromatic Amino Acids on Early/Structural Osteoclast Markers

**Fig. 1:**
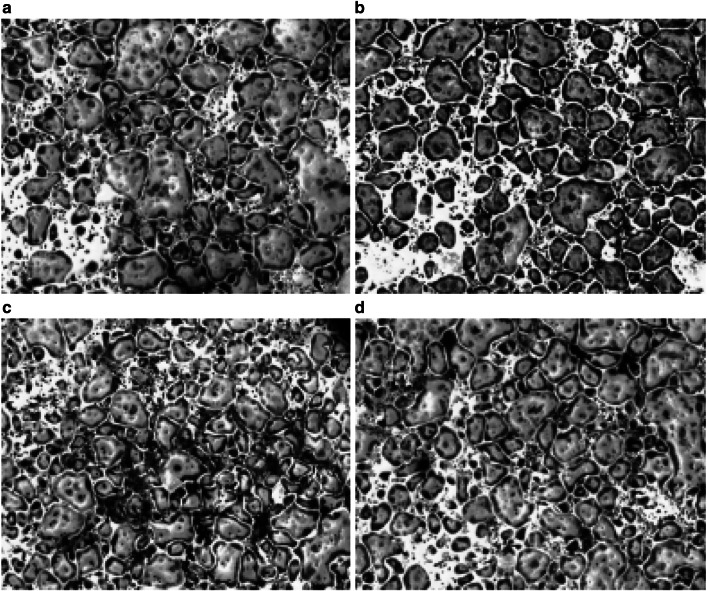
TRAP staining of bone marrow macrophages. Bone marrow macrophages were collected and spun at 1300 rpm for 8 min at room temperature, 660,000 cells were seeded in each well of the six-well plate with M-CSF (20 ng/ml) and RANKL (100 ng/ml). Shown is a representative picture of TRAP staining repeated at least three different times, **a** TRAP staining after 6 days of differentiation; **b** TRAP staining of osteoclasts incubated for the last 3 days with 100 µM tryptophan. **c** TRAP staining of osteoclasts incubated for the last 3 days with 100 µM phenylalanine. **d** TRAP staining of osteoclasts incubated for the last 3 days with 100 µM Tyrosine. Photomicrographs showed multinucleated osteoclastic cells in the control and the treated groups and of note; no changes were detected in the morphology of the cells

**Fig. 2:**
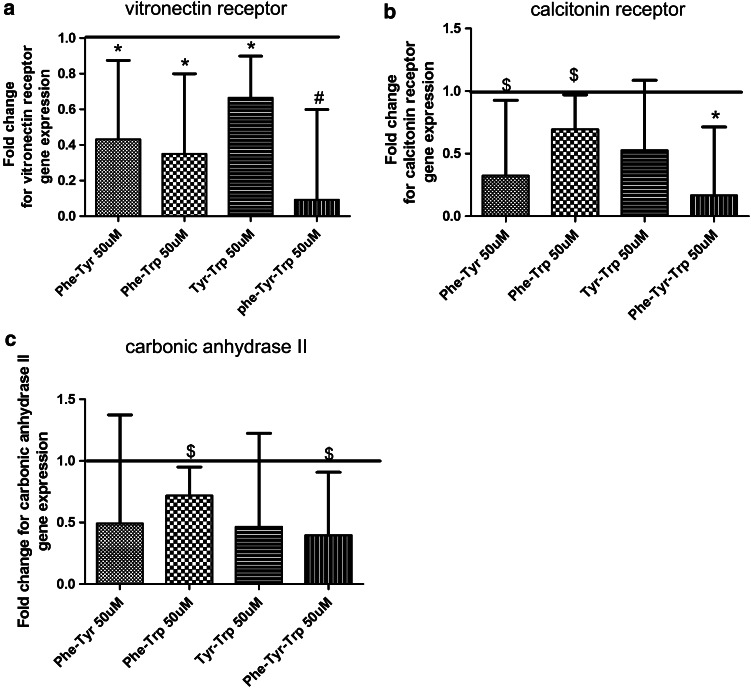
Effects of different aromatic amino acid combinations on vitronectin receptor, calcitonin receptor, and carbonic anhydrase II gene expression. Bone marrow macrophages were collected and spun at 1300 rpm for 8 min at room temperature, 660,000 cells were seeded in each well of the six-well plate with M-CSF (10 ng/ml) and RANKL (50 ng/ml). Different aromatic amino acid combinations were used to treat the cells during osteoclastic differentiation at a 50 μM concentration. Untreated cells were used as a control. **a** Amino acid combinations that down-regulated vitronectin receptor gene expression. **b** AA treatment groups that down-regulated calcitonin receptor gene expression. **c** AA combinations that down-regulated carbonic anhydrase II gene expression. Untreated cells were used as control (fold change of expression = 1). Results are expressed as geometric mean and geometric SEM for at least three independent experiments. *p ≤ 0.05, # *p* ≤ 0.01 and $ < 0.1

**Fig. 3:**
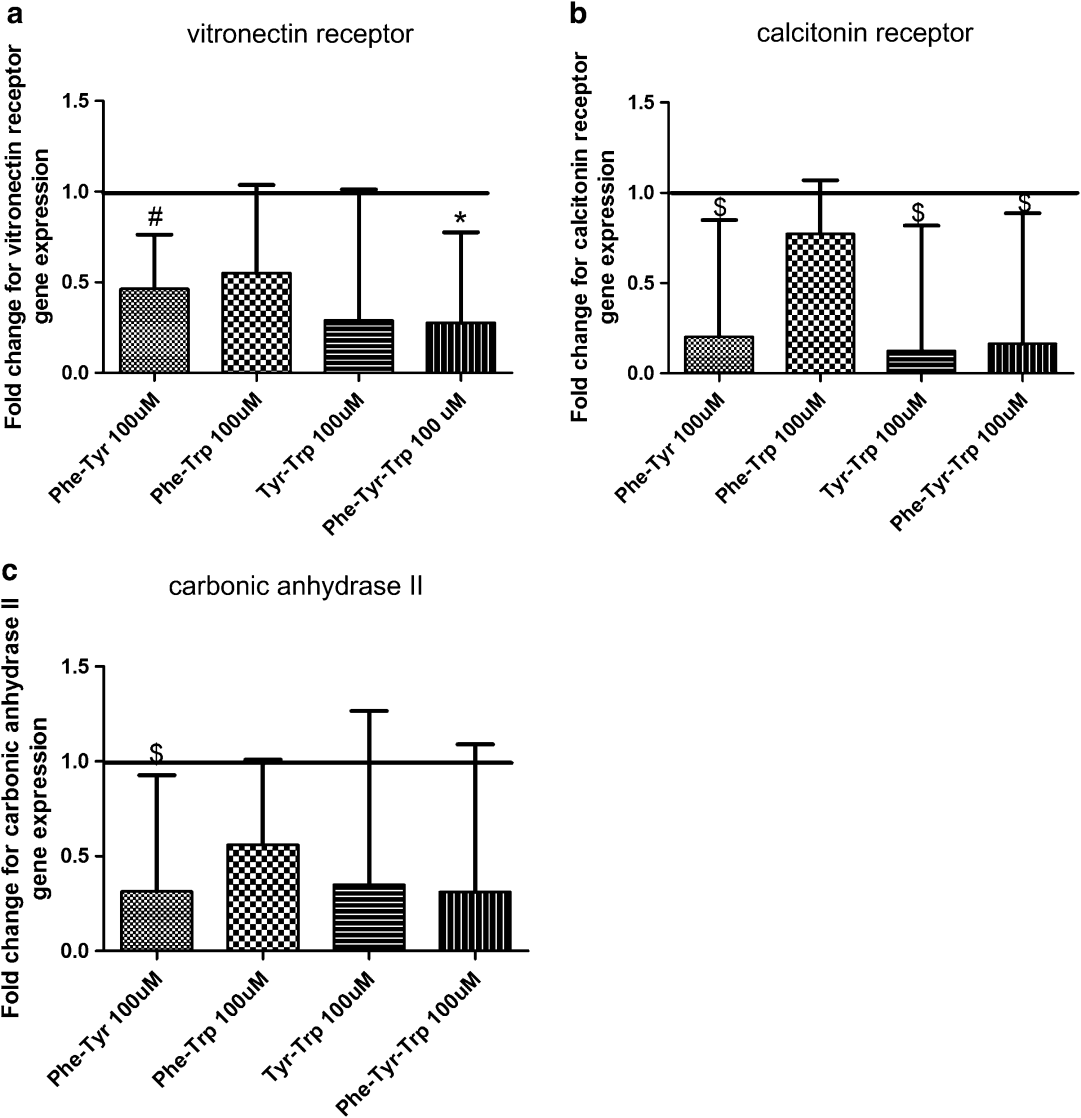
Effects of different aromatic amino acid combinations on vitronectin receptor, calcitonin receptor, and carbonic anhydrase II gene expression. Various aromatic amino acid combinations were used to treat the cells during osteoclastic differentiation at a 100 μM concentration and untreated cells were used as a control. **a** Amino acid combinations that down-regulated vitronectin receptor gene expression. **b** Different treatment groups that down-regulated calcitonin receptor gene expression. **c** Different amino acids that down-regulated carbonic anhydrase II gene expression. Untreated cells were used as control (fold change of expression = 1).Results are expressed as geometric mean and geometric SEM for at least three independent experiments. *p ≤ 0.05, # *p* ≤ 0.01, and *p* < 0.1

The effects of aromatic amino acid combinations on the early osteoclastic gene markers, vitronectin receptor, calcitonin receptor, and carbonic anhydrase II were first examined. Before extracting the RNA, osteoclast identity was confirmed by TRAP staining (Fig. 1). Aromatic amino acids (50 and 100 μM) down-regulated vitronectin gene expression in the TRAP stained cells. At the 50 µM concentration, the AA combinations of phenylalalanine and tyrosine, phenylalanine and tryptophan, tyrosine and tryptophan and phenylalanine-tyrosine-tryptophan all significantly decreased the gene expression of vitronectin receptor with a *p* value ≤ 0.05 as shown in Fig. 2a. At the 100 μM concentration, the AA combinations of phenylalanine-tyrosine and phenylalanine-tyrosine-tryptophan similarly significantly decreased the expression of vitronectin receptor gene expression (Fig. 3a).

For the calcitonin receptor gene expression, the aromatic amino acids combination of phenylalanine-tyrosine, phenylalanine-tryptophan, and phenylalanine-tyrosine-tryptophan in a 50 μM concentration down-regulated the gene expression as in Fig. 2b. A similar trend of gene expression down-regulation was observed at 100 μM concentration in the following AA combinations; phenylalanine-tyrosine, tyrosine-tryptophan, and phenylalanine-tyrosine-tryptophan (Fig. 3b).

For carbonic anhydrase II, the AA combinations of phenylalanine-tryptophan and phenylalanine-tyrosine-tryptophan (50 µM) decreased its gene expression as shown in Fig. 2c. In contrast at 100 μM only the phenylalanine-tyrosine combination showed a trend of down-regulation (Fig. 3c).

### Effect of Aromatic Amino Acids on Late/Functional Markers of Osteoclasts

**Fig. 4:**
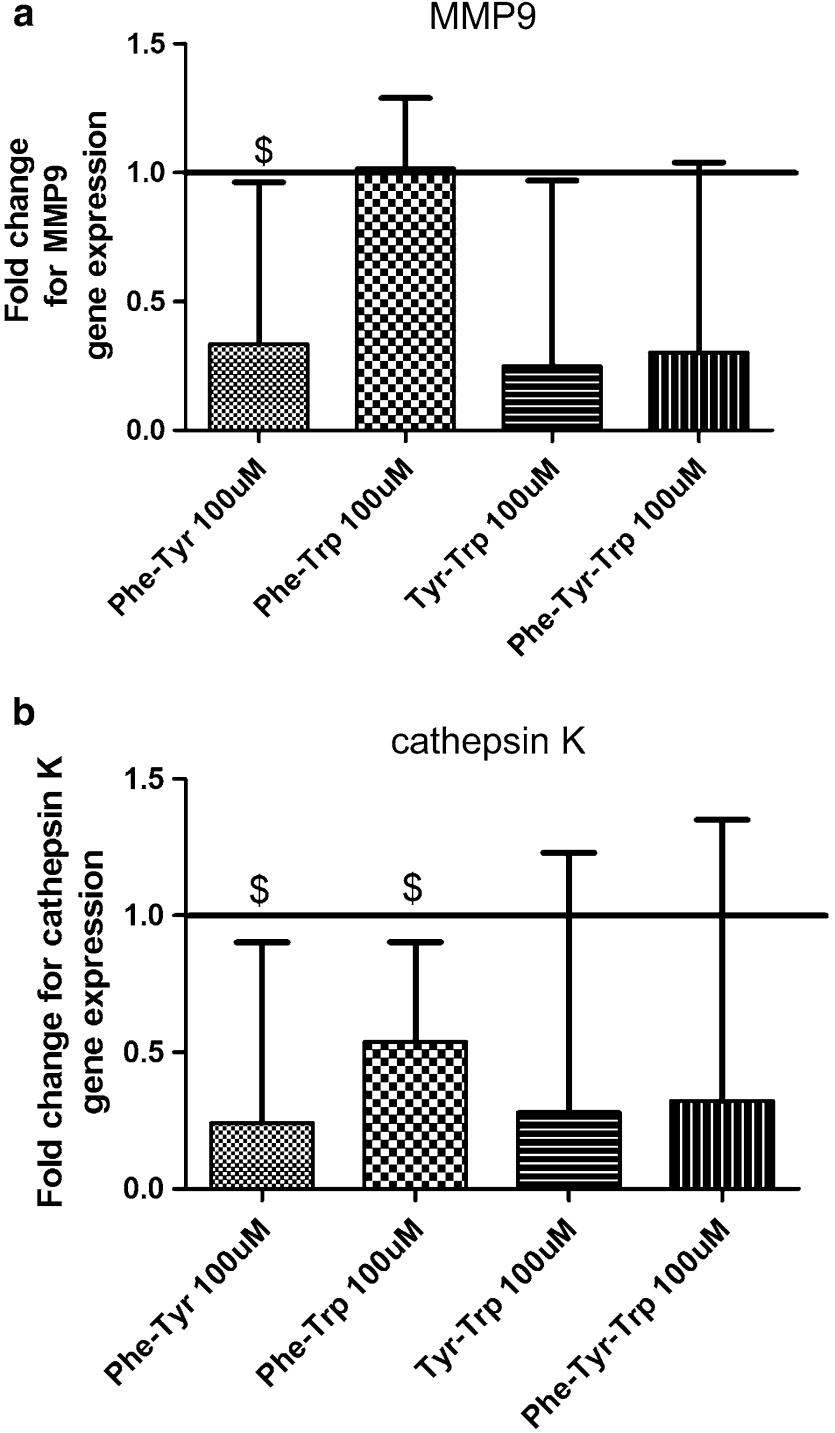
Effects of different aromatic amino acids on matrix metalloproteinase 9, cathepsin K gene expression. Isolated bone marrow macrophages: **a** Amino acid combinations (50 μM) down-regulated matrix metalloproteinase 9 gene expression. **b** AA treatment groups (50 μM) that down-regulated cathepsin K gene expression. Untreated cells were used as a control (fold change of expression = 1). Results are expressed as geometric mean and geometric SEM for at least three independent experiments. $ < 0.1

At the AA concentration of 100 μM the AA combinations of phenylalanine-tyrosine, tyrosine-tryptophan and phenylalanine-tyrosine-tryptophan all down-regulated MMP-9 expression although none of these changes reached statistical significance (Fig. 4a).

For cathepsin K (50 μM), the AA combinations of tyrosine-tryptophan and phenylalanine-tyrosine-tryptophan down-regulated its gene expression. At the higher AA concentration (100 μM), the combinations of phenylalanine-tyrosine and phenylalanine-tryptophan down-regulated cathepsin K gene expression (Fig. 4b).

### Effect of Aromatic Amino Acids on Osteoclastic Activity

**Fig. 5:**
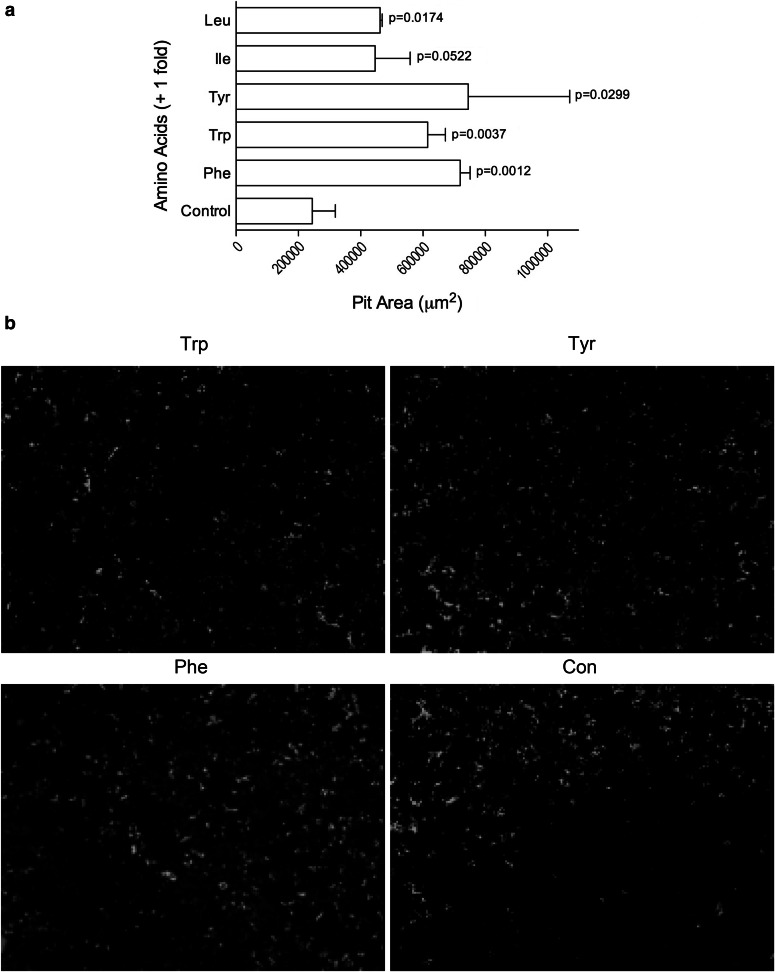
Effects of different amino acids on osteoclastic resorption activity. **a** Bone marrow macrophages were cultured in 16 well BD BioCoat Osteologic slides and stimulated with the indicated amino acid for 3 days. Resorption pits were visualized by Von Kossa staining. Pit area was estimated using NIH Image J. Results are expressed as mean ± SEM for at least three independent experiments. Significance value is listed above each graph *bar*. **b** Aromatic amino acids (Phe, Tyr and Trp) showed more resorption pits compared to the control

To evaluate the impact of aromatic amino acids on osteoclastic activity, we used the pit resorption assay (Fig. 5). Isolated bone marrow macrophages were differentiated into osteoclasts in the presence of M-CSF and RANK-L and exposed to aromatic amino acids (tyrosine, tryptophan, or phenylalanine) or leucine or isoleucine. All the aromatic amino acids significantly increased resorption activity. Non aromatic amino acids leucine and isoleucine also increased resorptive activity but to a lesser extent.

## Discussion

In present study, we examined the effects of aromatic amino acids on osteoclast differentiation as our previous data showed that aromatic amino acid supplementation prevented bone loss in aging C57BL/6 mice placed on a low protein diet [15]. Other studies [31, 32] by Hampson et al. [31] showed that nutritional supplementation (12–24 g protein, 12–24 g fat, and 37–74 g carbohydrate together with vitamins, minerals, and trace elements daily) in elderly women over a 1 year interval showed a reduction in serum osteoprotegerin and serum C-terminal telopeptide of type I collagen by ∼30% and showed an increase in bone alkaline phosphatase and osteocalcin and glucose has been reported to affect osteoclastic activity as Wittrant et al. [33] showed the first evidence that high D-glucose inhibited RANKL-mediated signaling events that correlated to osteoclast differentiation and function. However, the effect of aromatic amino acids on osteoclasts had not previously been examined. The C57BL/6 mouse model was used due to consistent results from these mice with published data from human clinical trials and because it is a model of aging as previously characterized by our group [34]. Aromatic amino acids down-regulated vitronectin gene expression and alternatively osteoclast differentiation and polarization as vitronectin gene is crucial for osteoclast polarization into clear zones and ruffled borders; two of the most characteristic features of osteoclasts [3]. Our results also demonstrated that aromatic amino acids down-regulated early osteoclast differentiation by suppressing calcitonin receptor gene expression as the CTR is a G protein-coupled surface receptor that is expressed at high levels by osteoclast and binds with highest affinity to calcitonin that regulates calcium homeostasis [5]. Real time PCR data showed a decrease in carbonic anhydrase II gene expression, an early marker of osteoclast differentiation and important for bone resorption as it facilitates proton production and thus the acidic environment of the resorption lacunae [7].

MMP-9 and cathepsin K are considered late markers of osteoclastic differentiation as studies showed that NFATc1 plays a key role in up-regulating expression of genes required for osteoclast maturation, such as TRAP [35], cathepsin K[36], or MMP-9[37], which are crucial for bone resorption mediated by mature osteoclasts. Our data demonstrate a down-regulation in MMP-9 and cathepsin K as MMP-9 is crucial in bone turnover and remodeling [10] and cathepsin K is a major cysteine protease expressed in osteoclasts [11] and has a critical role in osteoclastic bone resorption. However, our TRAP staining photomicrographs showed no changes in the morphology or the number of nuclei of the cells in response to the aromatic amino acid treatment and these are consistent with PCR data that showed most effects on the attachment proteins.

Unexpectedly aromatic amino acids increased in vitro resorptive activity (Fig. 5). These data would suggest that aromatic amino acids main suppressive effect on osteoclasts may be through modulation of their attachment since resorptive activity was actually increased in the mature osteoclasts.

In conclusion, our data demonstrate that aromatic amino acids downregulate early and late osteoclast differentiation markers thus may contribute through this mechanism to the net increase in bone mass seen in vivo. Our data also suggest a link between the attachment protein; vitronectin receptor and the secreted cathepsin K that both showed consistent effects to the amino acid treatment. However, the non-attachment related proteins, carbonic anhydrase II, demonstrated less consistent effects in response to treatment.
